# Supplementary figures and images for: VSTM1-v2 does not drive human Th17 cell differentiation: A replication study
Source: PLoS One. 2023 Apr 13;18(4):e0284404. doi: 10.1371/journal.pone.0284404 (PMC10101491; doi:10.1371/journal.pone.0284404)

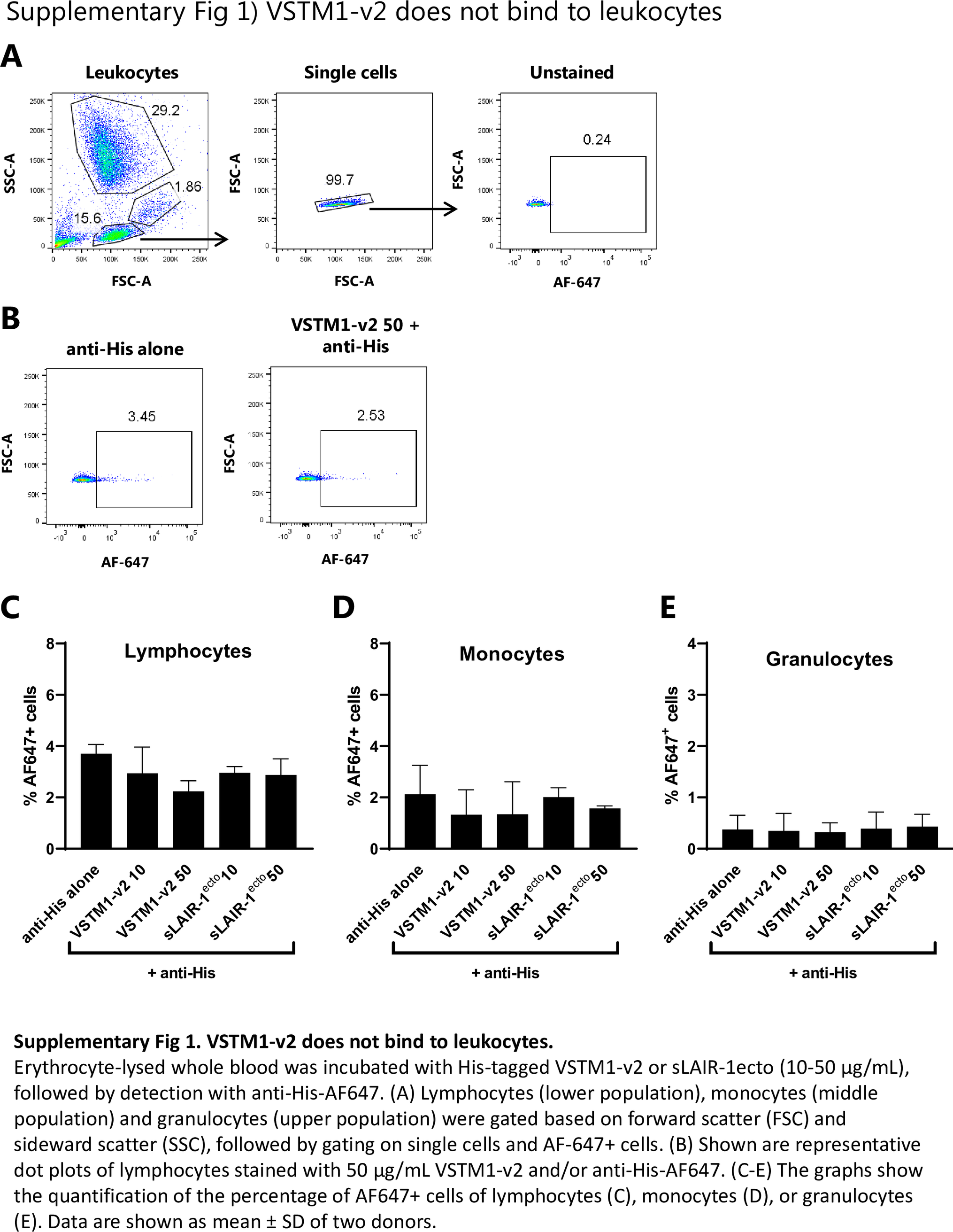

Supplement: S1 Fig — Erythrocyte-lysed whole blood was incubated with His-tagged VSTM1-v2 or sLAIR-1ecto (10–50 μg/mL), followed by detection with anti-His-AF647. (A) Lymphocytes (lower population), monocytes (middle population) and granulocytes (upper population) were gated based on forward scatter (FSC) and sideward scatter (SSC), followed by gating on single cells and AF-647+ cells. (B) Shown are representative dot plots of lymphocytes stained with 50 μg/mL VSTM1-v2 and/or anti-His-AF647. (C-E) The graphs show the quantification of the percentage of AF647+ cells of lymphocytes (C), monocytes (D), or granulocytes (E). Data are shown as mean ± SD of two donors. (TIF) [file pone.0284404.s001.tif]
