# Supplementary material for: VSTM1-v2 does not drive human Th17 cell differentiation: A replication study
Source: PLoS One. 2023 Apr 13;18(4):e0284404. doi: 10.1371/journal.pone.0284404 (PMC10101491; doi:10.1371/journal.pone.0284404)
Supplement: Attachment — Submitted filename: Response to Reviewers.docx [file pone.0284404.s002.docx]

Manuscript reference number: PONE-D-22-25685

Dear Editor,

We thank you for the evaluation of our manuscript entitled “VSTM1-v2 does not drive human Th17 cell differentiation: a replication study”.

We have addressed the reviewers’ comments and performed the requested experiment to increase the number of replicates. You can find our point-by-point reply to the reviewers’ comments below. Changes in the original manuscript are indicated as tracked changes.

We hope that, with these changes, you will find the manuscript acceptable for publication in *PLOS ONE.*

Our funding statement is as follows:
“This work was supported by Amsterdam UMC, University of Amsterdam, and by a Vici grant from the Netherlands Organization for Scientific Research (NWO, grant no. 91815608, [www.nwo.nl](https://eur04.safelinks.protection.outlook.com/?url=http%3A%2F%2Fwww.nwo.nl%2F&data=05%7C01%7Cf.m.hafkamp%40amsterdamumc.nl%7Cbeeaa37d0214449f07fc08dac8b309ea%7C68dfab1a11bb4cc6beb528d756984fb6%7C0%7C0%7C638042968709949580%7CUnknown%7CTWFpbGZsb3d8eyJWIjoiMC4wLjAwMDAiLCJQIjoiV2luMzIiLCJBTiI6Ik1haWwiLCJXVCI6Mn0%3D%7C3000%7C%7C%7C&sdata=5RMSUPV5EO6i4yEtqhkrbiq0GC0JOs2Ty4KnbppXhFU%3D&reserved=0) ). The funders had no role in study design, data collection and analysis, decision to publish, or preparation of the manuscript."

Yours sincerely,

Helen von Richthofen

Linde Meyaard

Reviewer #1: For figure 1B, n=7 seems too low for providing conclusive results. few more samples should be included ( n=20 fir example).
Representative Dot plot for IL17+ cells should be included at least as supporting figures.
ELISA of IL17 from Naive and memory T cells after treatment ( for cytokine induced Th17 differentiation ) could be considered

As requested by reviewer #1, we increased the number of replicates to strengthen the conclusion that VSTM1-v2 does not enhance DC-driven Th17 cell development from human naive T cells. Addition up to n=20 was not feasible as per experiment this takes approximately three weeks, but we did add n=3 to the figure with now a total of n=10. Statistical analysis still shows that no significant induction of Th17 cells occurs by VSTM1-v2. Furthermore, we included representative dot plots for IL-17^+^ cells as panel B in Figure 1.

We thank reviewer #1 for the suggestion to consider an ELISA to measure IL-17 in supernatant as a measure for Th17 cell differentiation. In our previous publication by Souwer *et al.* [1], we also assessed DC-driven Th17 differentiation with or without neutrophils, and we measured a similar fold induction in % IL-17+ cells versus IL-17 concentration in supernatant. Thus, these methods seem equally sensitive. However, measuring the % of IL-17+ cells also gives single cell information as opposed to using ELISA, which is why we chose this method.

Reviewer #2: The manuscript provides a controversial opinion on the role of VSTM1-v2 in human Th17 cell differentiation against a previous study. To draw a clear conclusion, the author should supply additional data to answer the following questions:

1) The VSTM1-v2 expression construct has not be verified by sequencing therefore, the protein generated using this construct may not be correct.
We did sequence the VSTM1-v2 construct as part of our standard cloning procedure, and confirmed that the sequence is correct. The sequence can be found in our recent publication in J Immunol (Supplemental Figure 1) [2].

2) It is not clear whether VSTM1-v2 interacts with a ligand used by the full length SIRL-1.
We did not detect direct binding between SIRL-1 and its ligands (S100 proteins, LL-37 and phenol soluble modulins) [3, 4], possibly due to low affinity interactions or the requirement of an additional binding partner. As a consequence, we were unfortunately not able to technically assess whether VSTM1-v2 competes with the binding between SIRL-1 and its ligands. We now clarified this in sentence 168-170 of the manuscript.

3) Since purified VSTM1-v2 may lose function, overexpression of this protein may be more feasible.
We appreciate the suggestion of the reviewer, but we purposefully chose to use purified recombinant VSTM1-v2 for several reasons; 1) we aimed to replicate the study by Guo *et al*., in which purified VSTM1-v2 was used; 2) purification of recombinant proteins by His-tag is an approved and commonly used method to obtain functional recombinant proteins [5], and 3) we made use of primary cells in this system, which are technically challenging to transfect for overexpression.

References

1.	Souwer Y, Groot Kormelink T, Taanman-Kueter EW, Muller FJ, van Capel TMM, Varga DV, et al. Human TH17 cell development requires processing of dendritic cell-derived CXCL8 by neutrophil elastase. The Journal of allergy and clinical immunology. 2018;141(6):2286-9 e5.

2.	von Richthofen HJ, Westerlaken GHA, Gollnast D, Besteman S, Delemarre EM, Rodenburg K, et al. Soluble Signal Inhibitory Receptor on Leukocytes-1 Is Released from Activated Neutrophils by Proteinase 3 Cleavage. J Immunol. 2023.

3.	Rumpret M, von Richthofen HJ, van der Linden M, Westerlaken GHA, Talavera Ormeno C, Low TY, et al. Recognition of S100 proteins by Signal Inhibitory Receptor on Leukocytes-1 negatively regulates human neutrophils. Eur J Immunol. 2021;51(9):2210-7.

4.	Rumpret M, von Richthofen HJ, van der Linden M, Westerlaken GHA, Talavera Ormeno C, van Strijp JAG, et al. Signal inhibitory receptor on leukocytes-1 recognizes bacterial and endogenous amphipathic alpha-helical peptides. FASEB J. 2021;35(10):e21875.

5.	Spriestersbach A, Kubicek J, Schafer F, Block H, Maertens B. Purification of His-Tagged Proteins. Methods Enzymol. 2015;559:1-15.
