# Supplementary material for: VSTM1-v2 does not drive human Th17 cell differentiation: A replication study
Source: PLoS One. 2023 Apr 13;18(4):e0284404. doi: 10.1371/journal.pone.0284404 (PMC10101491; doi:10.1371/journal.pone.0284404)
Supplement: Attachment — Submitted filename: Rebuttal.docx [file pone.0284404.s003.docx]

Manuscript reference number: PONE-D-22-25685

Dear Dr Sumit Kumar Hira,

Thank you for your evaluation of the revision of our manuscript entitled “VSTM1-v2 does not drive human Th17 cell differentiation: a replication study” for *PLOS ONE*.

Both reviewers now indicate that all their comments were addressed in the revised manuscript, yet your recommendation is to revise the manuscript again. We assume this is because of reviewer #2’s comment to question 6, suggesting to use supernatants of VSTM1-v2-transfected cells instead of purified VSTM1-v2 for our experiments.

In the first round of review, reviewer #2 also raised this concern. We replied to this comment in the following way: “… we purposefully chose to use purified recombinant VSTM1-v2 for several reasons; 1) we aimed to replicate the study by Guo *et al*., in which purified VSTM1-v2 was used; 2) purification of recombinant proteins by His-tag is an approved and commonly used method to obtain functional recombinant proteins, and 3) we made use of primary cells in this system, which are technically challenging to transfect for overexpression.”

These arguments still stand, we purposely chose to use purification of His-tagged recombinant VSTM1-v2 with a Ni-Sepharose column in order to replicate the method used by Guo *et al*. Additionally, cell culture supernatant contains a plethora of compounds which may have indirect effects on the co-culture system that we use.

We characterized VSTM1-v2 by SDS-PAGE and Western blot after purification (von Richthofen et al., 2023). VSTM1-v2 had a molecular weight of approximately 50 kDa, and deglycosylated VSTM1-v2 had a molecular weight of 37 kDa. This is in accordance with the findings by Guo *et al.*. Moreover, we could detect native VSTM1-v2 with anti-SIRL-1 antibodies (two clones), which is to be expected since VSTM1-v2 is a splice isoform of SIRL-1. This suggests that purification of VSTM1-v2 resulted in a viable protein. We did not describe the characterization of VSTM1-v2 protein in the current manuscript, since we already described it in our earlier publication. However, to avoid confusion, we now added the following sentence to the discussion, after sentence 175: “Purified VSTM1-v2 had a similar molecular weight as in the study by Guo *et al*., as assessed by SDS-PAGE (von Richthofen et al., 2023). “

Taken together, we conclude that using supernatants of VSTM1-v2 transfected cells rather than purified VSTM1-v2 will not improve the quality of the manuscript. We therefore hope that with this addition the manuscript is acceptable for publication in *PLOS ONE* .

Yours sincerely,

Helen von Richthofen
Linde Meyaard

von Richthofen, H. J., Westerlaken, G. H. A., Gollnast, D., Besteman, S., Delemarre, E. M., Rodenburg, K., . . . Meyaard, L. (2023). Soluble Signal Inhibitory Receptor on Leukocytes-1 Is Released from Activated Neutrophils by Proteinase 3 Cleavage. *Journal of Immunology, 210*(4), 389-397. doi:10.4049/jimmunol.2200169
